# Depletion of T-cell intracellular antigen proteins promotes cell proliferation

**DOI:** 10.1186/gb-2009-10-8-r87

**Published:** 2009-08-26

**Authors:** Raquel Reyes, José Alcalde, José M Izquierdo

**Affiliations:** 1 Centro de Biología Molecular 'Severo Ochoa', Consejo Superior de Investigaciones Científicas-Universidad Autónoma de Madrid, C/Nicolás Cabrera 1, Lab-107, Cantoblanco DP 28049, Madrid, Spain

## Abstract

**Background:**

T-cell intracellular antigen-1 (TIA-1) and TIA-1 related/like protein (TIAR/TIAL1), two DNA/RNA binding proteins broadly expressed in eukaryotic cells, participate in the regulation of gene expression through RNA metabolism. Despite the biological relevance of these regulators, there are no genome-wide studies assessing global transcriptomic and phenotypic impacts after changes in the expression and/or function of these proteins.

**Results:**

Using high-throughput gene expression profiling, we found that the TIA-1/TIAR-depleted cell phenotype is linked to a transcriptome involved in the control of inflammation, cell-cell signaling, immune-suppression, angiogenesis, metabolism and cell proliferation. Induced genes included pro-inflammatory cytokines, inflammatory chemokines, growth-stimulating factors and pro-angiogenic inducers. Repressed genes involved the RAS oncogene family member RAB40B, regulators of cytoskeleton organization and biogenesis and a mitochondrial modulator. Consistent with these observations, depletion of TIA proteins in HeLa cells results in increased cell proliferation, altered cell-cycle and anchorage-independent growth. Mechanistically, the changes associated with the steady-state target mRNA levels regulated by TIA proteins are consistent with overlapping effects on gene basal transcription rate and mRNA turnover.

**Conclusions:**

Collectively, our findings suggest a role for TIA proteins as cellular sensors that modulate gene expression control at the transcriptional and post-transcriptional levels, coupling cell proliferation responses and metabolic homeostasis to cell survival and growth.

## Abstract 2

The transcriptome of TIA-1/TIAR-depleted cells indicates roles in inflammation, cell-cell signaling, immune suppression, angiogenesis, metabolism and cell proliferation.

## Background

One of the most important challenges resulting from our knowledge of the human genome - and, in general, of all genomes - is to understand how the collection of RNAs and proteins that define us as an organism are generated. Nowadays, we accept that the origin of our transcriptomic and proteomic diversity is due not just to the number of genes present in our genome but also to the dynamic and differential regulation of their expression. The characterization of transcriptional and post-transcriptional events leading to the generation of multiple RNAs, proteins and functions from a single or distinct RNA precursors reveals the existence of multiple layers and networks involved in regulating the diverse biological functions controlled by the transcriptome [1].

T-cell intracellular antigen (TIA)-1 and TIA-1 related/like factor (TIAR/TIAL1) are two proteins that play important roles in many aspects of RNA biology, from RNA transcription to splicing, stability and translation. Indeed, TIA proteins have been shown to interact with RNA polymerase II [2], DNA [3,4] and RNA [5] and to participate in the control of alternative pre-mRNA splicing [6-8]. TIA proteins bind to uridine-rich sequences, which are mostly located in the introns, and seem to facilitate the recruitment of U1 small nuclear ribonucleoprotein, thus promoting the recognition and processing of atypical 5' splice sites [9,10]. The cytoplasmic pool of these proteins has been linked to the control of translation and/or stability of some mRNAs through binding to adenine- and uridine-rich sequences located in 3' untranslated regions [11-14]. Additionally, these proteins are involved in cell responses to metabolic and genotoxic stresses as well as the formation of stress granules [15,16]. It has also been reported that these proteins play a relevant role in the control of cell death [17-19] and viral replication [20]. In fact, mice that lack either TIA-1 or TIAR show high rates of embryonic lethality, indicating a relevant role for these proteins during embryonic development [12,21]. Furthermore, recent studies have identified a set of mRNAs that interact and are regulated by these proteins [22-24].

Altogether, these findings strongly support a key role for TIA-1 and TIAR proteins in the control of different aspects of RNA metabolism and function. However, little is known about how these regulators control the transcriptome and about the specific and/or overlapping functions associated with TIA proteins at this level. Here, we use a loss-of-function approach combined with global mRNA expression pattern and phenotypic profiling analyses to identify targets and pathways regulated by TIA proteins. Our data suggest that these proteins are involved in the transcriptional and post-transcriptional regulation of specific transcripts associated with the control of key cellular functions, such as inflammation, cell proliferation and anchorage-independent growth responses.

## Results

### Gene expression profiling of TIA-1 and TIAR-depleted HeLa cells

**Figure 1:**
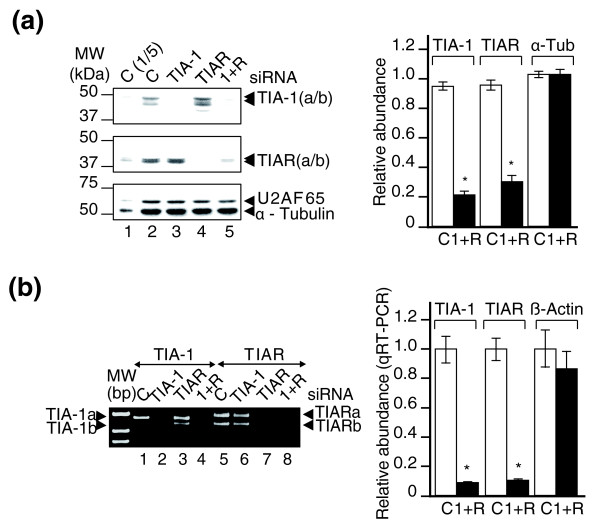
RNA interference-mediated depletion of TIA proteins in HeLa cells. **(a) **Immunoblot analysis of HeLa cell lysates (5 μg (lane 1; C(1/5)) or 25 μg (lanes 2 to 5)) prepared 72 h after transfection with siRNAs against control (C; lanes 1 and 2), TIA-1 (lane 3), TIAR (lane 4), and TIA-1 plus TIAR (lane 5; 1+R). TIA-1(a/b) and TIAR(a/b) refer to the major isoforms of TIA-1 and TIAR, respectively. The blot was probed with antibodies against TIA-1, TIAR, U2AF65 and α-tubulin proteins, as indicated. Molecular weight markers and the identities of protein bands are shown. The intensities of the different protein bands from immunoblots were quantified by densitometry. The represented values were normalized and are expressed relative to α-tubulin (α-Tub), whose value is fixed arbitrarily to 1, and are means ± standard error of the mean (SEM; n = 10; *P < 0.05). **(b) **Cytoplasmic mRNAs from post-transfected HeLa cells in (a) were analyzed by RT-PCR. Positions of size markers and the predicted alternatively spliced products are indicated. Quantification of relative levels of TIA-1, TIAR and β-actin mRNAs in the above post-tranfected HeLa cells by real time RT-PCR. The represented values were normalized and are expressed relative to β-actin mRNA, whose value is fixed arbitrarily to 1, and are means ± SEM (n = 3; *P < 0.01).

To study the role of TIA-1 and TIAR in the global control of gene expression, we transfected HeLa cells with double-stranded small interfering RNAs (siRNAs) targeting TIA-1 and/or TIAR mRNAs or with control siRNA (c) as previously described [10,25-27]. The effect of the siRNAs on TIA-1 and TIAR expression was assessed by western blotting (Figure 1a) and by semiquantitative and quantitative RT-PCR analyses (Figure 1b). Under these experimental conditions, 80 to 90% depletion of both TIA-1 and TIAR proteins (Figure 1a) and mRNAs (Figure 1b) was achieved 72 h after transfection, in agreement with previous findings [10,25-27]. In contrast, U2AF65 and α-tubulin proteins (Figure 1a) and β-actin mRNA (Figure 1b), used as controls for siRNA specificity, were not significantly affected by the treatment.

**Figure 2:**
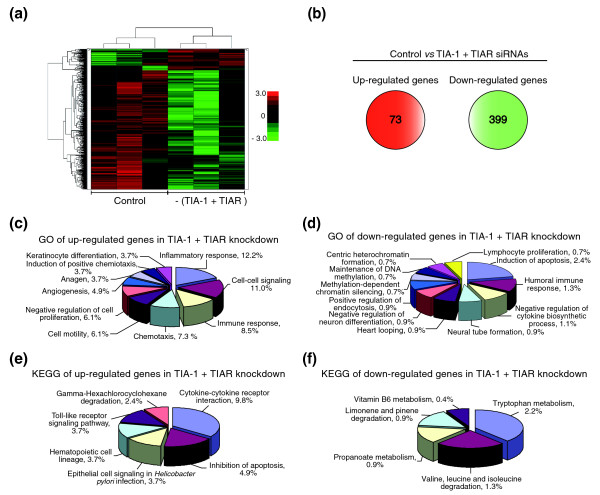
Knockdown of TIA proteins activates an acute inflammation-promoting transcriptome. **(a) **Heat map representation depicting microarray data for genes that are differentially regulated by siRNA-mediated reduction of TIA-1 and TIAR. Mean fold-change values from three independent replicates for each biological condition tested are given for 538 probe sets (horizontal lanes; out of a total of 47,400 sets) that were detectable above background showing at least a 1.5-fold change in expression and were statistically different (*P *< 0.05) in the two experimental conditions tested. Red and green indicate up- and down-regulation, respectively, relative to control siRNA-transfected HeLa cells. The color scale to the right indicates the magnitude of the fold change (base 2 logarithm) for a particular transcript. **(b) **Total number of genes whose expression was up- or down-regulated in TIA-1 and TIAR-depleted cells. **(c, d) **Graphic pie representations of the distribution of up- (c) and down-regulated (d) genes (*P *< 0.01) using the GO biological process category. **(e, f) **Graphic pie representations of the distribution of up- (e) and down-regulated (f) genes (*P *< 0.05) using the KEGG pathway database. In all cases (c-f), percentages shown reflect the portion of total genes that are associated with the biological functions and pathways indicated.

In order to define gene expression profiles resulting from the suppression of TIA-1 and TIAR expression, we assessed the differences in global gene expression patterns in HeLa cells transfected with siRNAs targeting both TIA-1 and TIAR compared to HeLa cells transfected with control siRNA. Comparisons were made using hierarchical clustering analysis of gene expression patterns from three array independent experiments performed in three different biological samples for each experimental condition tested (Additional data file 1). As shown in Figure 2a, depletion of TIA-1 and TIAR resulted in a marked alteration in the usual expression patterns of genes. To identify cohorts of genes regulated by TIA proteins, the gene expression array dataset was analyzed using Robust Multichip Average [28] normalization followed by identification of differentially expressed genes using the Rank Products method [29]. In these analyses, only genes showing at least a 1.5-fold difference in expression compared to the control were considered. A total of 472 genes were differentially expressed (*P *< 0.05), of which 73 and 399 were up- and down-regulated, respectively (Figure 2b; Additional data file 2).

In an attempt to address the functional relevance of the observed changes in high-density DNA microarrays, Gene Ontology (GO) analyses were performed for the up- and down-regulated genes. GO analysis was able to identify the main categories that had significant representation (*P *< 0.01) of differentially expressed genes controlled by TIA proteins (Figure 2c, d; Additional data file 3). GO categories related to inflammation and immune responses, cell proliferation, cell-cell signaling, chemotaxis, cell motility, angiogenesis and anagen were among the enriched categories in up-regulated genes (Figure 2c). On the other hand, GO categories associated with apoptosis induction, humoral immune response, cell differentiation and chromatin modifications were particularly prevalent in down-regulated genes (Figure 2d).

In the same vein, Kyoto Encyclopedia of Genes and Genomes (KEGG) database analysis integrating individual components into unified pathways was used to identify the enrichment of specific pathway components in functionally regulated gene groups (Figure 2e, f; Additional data file 3). The results indicate that six KEGG pathways were significantly enriched (*P *< 0.05) in up-regulated genes, including cytokine-cytokine receptor interaction, apoptosis inhibition, epithelial cell signaling in *Helicobacter pylori *infection, hematopoietic cell lineage, Toll-like receptor signaling pathway and gamma-hexachlorocyclohexane degradation (Figure 2e; Additional data file 3), and five KEGG pathways were significantly enriched (*P *< 0.05) in down-regulated genes, involving several metabolic pathways such as tryptophan metabolism, amino acid degradation, propionate metabolism, limonene and pinene degradation and vitamin B6 metabolism (Figure 2f; Additional data file 3).

Taken together, these results suggest the activation of inflammation and proliferation-promoting pathways followed by alterations in apoptotic and immune responses as well as in cell signaling and metabolism that can mediate or modulate their own pathway or the cross-talk between pathways that account for the functional reprogramming of TIA-1/TIAR-depleted HeLa cells.

### Distinct and overlapping functions of TIA-1 and TIAR in the control of the transcriptome

To determine whether TIA-1 and TIAR have selective and specific effects on the control of the gene expression pathways described above, we next performed gene expression profiling analyses in HeLa cells in which either TIA-1 or TIAR was inactivated (Additional data files 4 and Additional data files 5). Inactivation of TIA-1 revealed 124 differentially expressed genes (*P *< 0.05), of which 36 and 88 were up- and down-regulated, respectively (Additional data file 4). On the other hand, knockdown of TIAR resulted in the differential expression (*P *< 0.05) of 94 genes, of which 23 and 71 were up- and down-regulated, respectively (Additional data file 5).

Next, GO biological process categories and KEGG pathway analyses were performed as before. A summary of GO annotations by biological process categories is provided in Additional data file 6a-d. GO categories (*P *< 0.01) associated with both TIA-1 (Additional data files 6a and Additional data files 7) and TIAR (Additional data files 6b and Additional data files 8) knockdown are related to inflammation and cell proliferation processes, including up-regulated genes in the following categories: cell-cell signaling, inflammatory response, chemotaxis, negative and positive regulation of cell proliferation, immune response, G-protein coupled receptor protein signaling pathway, cell adhesion, angiogenesis, cell motility, transduction and cell surface receptor mediated signaling. These results agree with the observations previously found in HeLa cells under TIA-1 and TIAR double silencing (Figure 2c), indicating that TIA-1 and TIAR are redundant in the control of these pathways. However, GO categories (*P *< 0.01) of down-regulated genes associated with the depletion of either TIA-1 (Additional data files 6c and Additional data files 7) or TIAR (Additional data files 6d and Additional data files 8) showed striking differences in the gene clusters. Indeed, whereas GO categories of down-regulated genes in TIA-1 knockdown cells are linked to genes involved in apoptosis induction, negative regulation of cytokine biosynthesis and metabolic responses (Additional data files 6c and Additional data files 7, GO categories of down-regulated genes in TIAR knockdown cells were limited to genes implicated in RNA processing (Additional data files 6d and Additional data files 8).

Results from KEGG pathway analysis (*P *< 0.05) are provided in Additional data file 6e-h. KEGG data for up-regulated genes in TIA-1 (Additional data files 6e and Additional data files 7) and TIAR (Additional data files 6f and Additional data files 8) knockdown cells are associated with genes related to cytokine-cytokine receptor interaction, apoptosis inhibition, and immune, chemotaxis, inflammatory, proliferative and metabolic responses, in agreement with the results described above (Additional data file 6a, b). On the other hand, KEGG data for down-regulated genes in TIA-1 knockdown cells are linked to vascular epidermal growth factor signaling pathway and citrate and reductive carboxylase cycles (Additional data files 6g and Additional data files 7), and KEGG data for TIAR knockdown cells are associated with several pathways related to the metabolism of amino acids, vitamins, fatty acids, glycerophospholipids and energy availability (Additional data files 6h and Additional data files 8), suggesting that both TIA-1 and TIAR can regulate specific aspects of cell metabolism.

Collectively, these results indicate that TIA-1 and TIAR regulate specific and overlapping aspects of the transcriptome, suggesting that their functional effects can be redundant, additive and/or independent, in agreement with previous findings [8,12,21,27].

### A molecular hallmark involving cytokines, chemokines and growth-stimulating factors is associated with the depletion of TIA proteins

**Figure 3:**
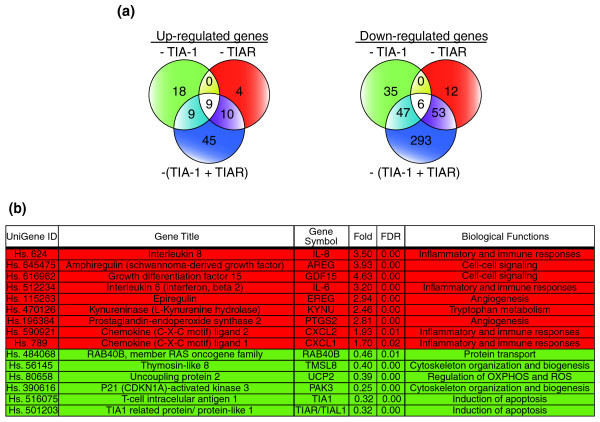
Identification of a gene cluster associated with the silencing of TIA proteins. **(a) **Venn diagrams depicting the numbers of genes that were up-regulated (left) or down-regulated (right) by TIA-1 (green), TIAR (red) or TIA-1 plus TIAR (blue) silencing. All data for all genes are presented in Additional data files 2 to 5 and 7 and 8. **(b) **Gene cluster defining a molecular signature of up- (highlighted in red) and down-regulated (highlighted in green) genes in TIA-1 and TIAR-depleted HeLa cells. The microarray data were analyzed by the Rank Products method [29]. Fold is an average measure of the fold change in differential expression and the false discovery rate (FDR) indicates the expected percentage of false positives. The Probe-set ID is the Unigene probe-set identifier [43].

To test whether reduced expression of TIA proteins is associated with the regulation of characteristic gene clusters, up- and down-regulated genes were distributed using Venn diagrams (Figure 3a). In these analyses, we identified a gene signature involving nine up- and six down-regulated genes (Figure 3a). This gene signature is composed of up-regulated genes, including *IL-8*, *AREG*, *GDF15*, *IL-6*, *EREG*, *KYNU*, *PTGS2*, *CXCL1 *and *CXCL2 *(Figure 3b, indicated in red) which are mediators of inflammation, cell-cell signaling, cell proliferation, tryptophan metabolism, immune and angiogenesis responses, and down-regulated genes such as *RAB40B*, *TMSL8*, *UCP2*, *PAK3*, *TIA-1 *and *TIAR *(Figure 3b, indicated in green), which represent a set of heterogeneous genes implicated in protein transport and phosphorylation, cytoskeleton organization and biogenesis, control of the production of reactive oxygen species/oxidative metabolism and induction of apoptosis.

### Validation of microarray-predicted changes in gene expression patterns

**Figure 4:**
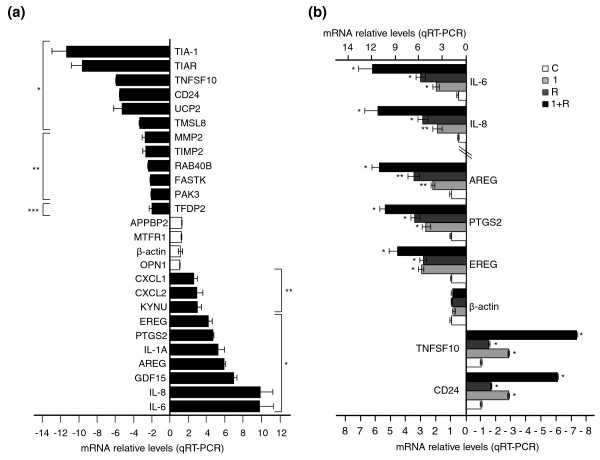
Validation of microarray-predicted changes by quantitative RT-PCR (qRT-PCR). **(a) **Differential expression of 26 genes, 10 up-regulated (bottom bars), 12 down-regulated (top bars) and 4 unchanged (middle bars), was verified by real time RT-PCR using specific primer pairs (Additional data file 11). The represented values were normalized and are expressed relative to β-actin, whose value is fixed arbitrarily to 1, and are means ± standard error of the mean (SEM; n = 3; *P < 0.001; **P < 0.01; ***P < 0.05). **(b) **Differential expression of eight genes, five up-regulated (*IL-6*, *IL-8*, *AREG*, *PTGS2 *and *EREG*), two down-regulated (*TNFSF10 *and *CD24*) and one unaffected (β-actin), from cytoplasmic RNA isolated from HeLa cells transfected with either control (white bars), TIA-1 (grey bars), TIAR (dark grey bars) or TIA-1 plus TIAR (black bars) siRNAs was verified as above. The represented values were normalized and are expressed relative to β-actin, whose value is fixed arbitrarily to 1, and are means ± SEM (n = 3; *P < 0.001; **P < 0.01).

The effects on steady-state mRNA levels detected by the microarray were independently validated using quantitative RT-PCR assays for 26 different genes. Figure 4a shows validation of predicted up-regulated (*EREG*, *PTGS2*, *IL-1A*, *IL-6*, *IL-8*, *AREG*, *GDF15*, *CXCL1*, *CXCL2 *and *KYNU*) and down-regulated (*TIA-1*, *TIAR*, *PAK3*, *TMSL8*, *UCP2*, *CD24*, *TNFSF10*, *RAB40B*, *MMP2*, *TIMP2*, *FASTK *and *TFDP2*) genes that were increased or decreased, respectively, by depletion of TIA-1 and TIAR in HeLa cells. As expected, the results confirmed predicted up- and down-regulation for all genes analyzed (Figure 4a). Furthermore, *APPBP2*, *MTFR1*, β-actin and *OPN1 *genes were used as controls (Figure 4a). Additionally, to know whether TIA-1 and TIAR have additive, overlapping or synergistic effects on steady-state target mRNA levels, some up-regulated (*IL-6*, *IL-8*, *AREG*, *PTGS2 *and *EREG*) and down-regulated (*TNFSF10 *and *CD24*) genes, as well as the β-actin gene as a control, were also validated by quantitative RT-PCR with independent silencing of either TIA-1 or TIAR. As shown in Figure 4b, depletion of TIA-1 or/and TIAR did affect partially the steady-state levels of analyzed mRNAs, showing that the actions of TIA proteins are additive and overlapping rather than synergistic. Taken together, these results are fully consistent with the observations performed by microarray analyses, suggesting that some changes observed in our microarray experiments could represent putative novel target genes regulated by TIA-1 and/or TIAR proteins.

### Depletion of TIA proteins alters both mRNA stability and basal gene transcription

**Figure 5:**
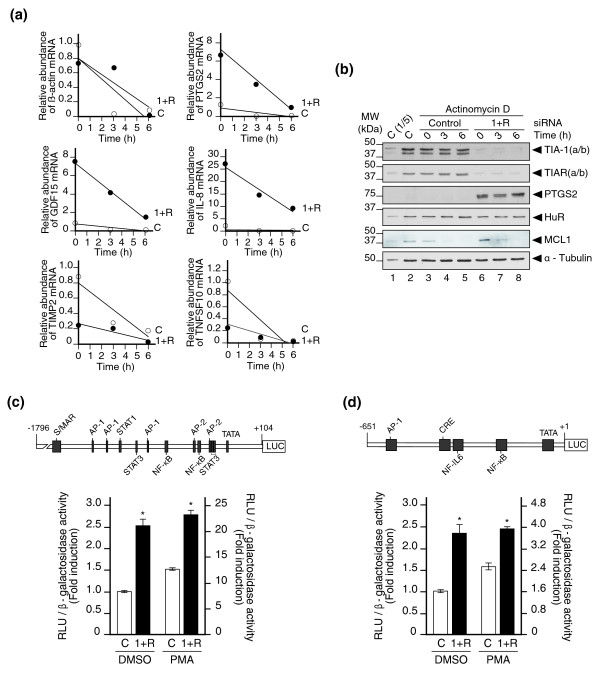
Silencing of TIA proteins alters both mRNA stability and gene transcription. **(a) **DNA transcription was inhibited in control and TIA-1/TIAR-depleted HeLa cells by the addition of actinomycin D (Act D; 5 μg/ml) to the culture medium. Cytoplasmic RNA was isolated at various times (0, 3 and 6 h) after the addition of the inhibitor Act D and analyzed by real time RT-PCR. Relative levels of β-actin, *PTGS2*, *GDF15*, *IL-8*, *TIMP2 *and *TNFSF10 *mRNAs were determined with the specific primer pairs (Additional data file 11). A representative experiment is shown. Steady-state RNA levels are represented at various times after the addition of Act D. Open and closed circles illustrate relative RNA levels in control (C) and TIA-1/TIAR (1+R)-depleted samples, respectively. **(b) **Validation of differential expression for selected genes by western blot analysis. Protein extracts (5 μg (lane 1; C(1/5)) or 25 μg (lanes 2 to 8)) derived from post-transfected HeLa cells with control (lanes 1 to 5) and TIA-1 and TIAR (lanes 6 to 8) siRNAs and treated with Act D as in (a) were analyzed with the indicated antibodies. A representative blot is shown. Molecular weight markers and the identities of protein bands are indicated. TIA-1(a/b) and TIAR(a/b) refer to the major isoforms of TIA-1 and TIAR, respectively. **(c, d) **Effect of the TIA-1/TIAR silencing on transcriptional activation of the *PTGS2 *(c) and *IL-6 *(d) gene promoters. A schematic representation of *PTGS2 *(c) and *IL-6 *(d) human gene promoters is shown. In both cases, *cis*-acting consensus sequences are represented by boxes. Control and TIA-1/TIAR-depleted HeLa cells were transiently cotransfected with *PTGS2 *or *IL-6 *promoter-driven firefly luciferase constructs together with a β-galactosidase-expressing plasmid (used as a transfection control) as described in Materials and methods. Sixteen hours after cotransfection, cells were cultured in the presence of DMSO or 20 ng/ml PMA for 6 h and assayed for luciferase and β-galactosidase activities. The represented values in (c, d) - the ratio between luciferase relative light units (RLU)/β-galactosidase activity - were normalized and are expressed relative to the control sample treated with DMSO, whose value is fixed arbitrarily to 1, and are means ± standard error of the mean (n = 3; *P < 0.001 in (c); *P < 0.01 in (d)).

To determine the mechanism underlying the alterations in gene expression observed through inactivation of TIA proteins, HeLa cells transfected with control siRNA or with TIA-1 and TIAR siRNAs were treated with actinomycin D, a selective inhibitor of RNA polymerase II (Figure 5a, b). The results suggest an effect on mRNA stability, as indicated by the steady-state mRNA levels of *PTGS2*, *GDF15*, *IL-8*, *TIMP2 *and *TNFSF10 *in TIA-1/TIAR-depleted HeLa cells (Figure 5a). However, the specific contribution of mRNA turnover can not be measured accurately in control HeLa cells because steady-state levels of some target mRNAs are extremely low. Perhaps a stressing stimulus that enhances mRNA decay is needed in order to establish properly whether TIA proteins affect mRNA stability. Additionally, some representative genes were also quantified at the protein level by western blotting. Figure 5b shows the results of TIA-1, TIAR and Prostaglandin-endoperoxide synthase 2 (PTGS2) protein expression levels, which are consistent with the real-time PCR results. As controls, Mcl-1 protein expression was specifically affected by the treatment with actinomycin D, but HuR and α-tubulin protein expression was not modified by either TIA-1 and TIAR depletion or by the treatment with actinomycin D (Figure 5b).

Given that the results on mRNA turnover do not conclusively explain the steady-state target mRNA levels in TIA-1/TIAR-depleted HeLa cells, we decided to estimate the contribution of the TIA-1/TIAR-induced silencing on transcription activation of two target genes. For this purpose, control and TIA-1/TIAR-depleted HeLa cells were cotransfected transiently with *PTGS2 *and *IL-6 *promoter-driven firefly luciferase constructs [30,31] and a β-galactosidase-expressing plasmid used as a transfection control. Sixteen hours after transfection, cells were stimulated with dimethyl sulfoxide (DMSO) or phorbol 12-myristate 13-acetate (PMA), as described in the Materials and methods section, and 6 hours after stimulation both luciferase and β-galactosidase activities were measured in whole-cell extracts. As shown in Figure 5c, d, the depletion of TIA proteins was able to promote a significant and reproducible induction (2- to 2.5-fold) of both luciferase constructs under the control of *PTGS2 *(Figure 5c) and *IL-6 *(Figure 5d) human promoter sequences, suggesting that reduced expression of TIA proteins deregulates the basal transcriptional activity of both promoters. Taken together, these results could suggest that the gene expression patterns detected in TIA-1/TIAR-depleted HeLa cells might be the result of an overlapping regulation, implying the involvement of several molecular events at the post-transcriptional and transcriptional levels.

### Silencing of TIA proteins results in increased cell proliferation and anchorage-independent growth

**Figure 6:**
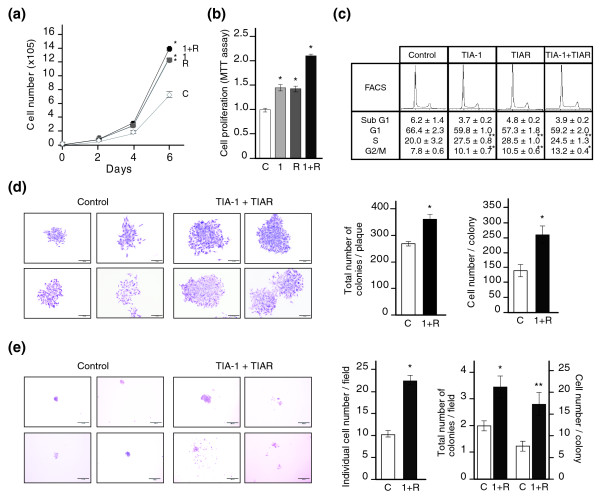
Depletion of TIA proteins activates cell proliferation and anchorage-independent growth. **(a) **Post-transfected HeLa cells with siRNAs against control (C; white diamond), TIA-1 (1; grey square), TIAR (R; grey circle) or TIA-1 plus TIAR (1+R; black circle) were seeded in six-well plates and the number was counted on the days indicated for 6 days. Each time point represents the means ± standard error of the mean (SEM; n = 3 to 6; *P < 0.01). **(b) **Cells grown for 6 days as in (a) were monitored by methyl thiazolyl tetrazolium (MTT) assays. The represented values were normalized and are expressed relative to control (C), whose value is fixed arbitrarily to 1, and are means ± SEM (n = 3 to 5; *P < 0.001). **(c) **Analysis of cell-cycle phases by flow cytometry after propidium iodide staining. The data are means ± SEM (n = 3; *P < 0.01; **P < 0.05). **(d) **Representative photographs of colonies generated from transfected HeLa cells. One week after seeding 2,000 cells in a 6-well plate, the cells were fixed in paraformaldehyde (5%), stained with crystal violet (0.01%) and quantified by light microscopy. The represented values are the means ± SEM from three independent experiments performed in duplicate for at least eleven independent colonies (*P < 0.001). A colony was defined as a cell cluster when containing at least 86 cells. Scale bars represent 200 μm. **(e) **Soft-agar colony formation. In each 6-well plate, 5,000 post-transfected HeLa cells as above were seeded into soft-agar matrix and incubated for 14 days. The cell number was counted under a light microscope after staining with crystal violet as before. The represented values are means ± SEM for three independent experiments performed in duplicate for at least 36 counted fields (*P < 0.001; **P < 0.01). In this case, a colony was defined as a cell cluster containing at least 4 cells. Scale bars represent 100 μm.

As shown in Figure 2 and in Additional data file 6, GO analyses suggested a role for TIA-1 and TIAR proteins in the control of cell proliferation. Thus, to validate at the functional level the results obtained with the GO analyses and to determine the putative role of TIA-1 and TIAR on cell proliferation, we examined the proliferative potential of HeLa cells with diminished expression of TIA-1 and/or TIAR. As shown in Figure 6, reduction of TIA-1 and/or TIAR expression in HeLa cells resulted in increased cell proliferation compared to control cells. Indeed, total cell numbers (Figure 6a) as well as measurement of metabolic activity by methyl thiazolyl tetrazolium assay (Figure 6b) support an individual and collective role for TIA proteins in the control of cell proliferation. Furthermore, TIA-1 and/or TIAR silencing promoted a significant increase in the proportion of cells in S and G2/M phases concurrent with a reduction in the proportion of cells in G0/G1 (Figure 6c). Most interestingly, the colony-forming capacity in plaque (Figure 6d) and soft agar (Figure 6e) was substantially increased in TIA-1/TIAR-depleted cells compared with the corresponding control cells. In addition, the number of colonies as well as the number of cells per colony were significantly increased in HeLa cells lacking TIA proteins (Figure 6d, e, left and right panels, respectively). Altogether, these results indicate that the knockdown of TIA-1 and TIAR proteins in HeLa cells triggers cell proliferation and anchorage-independent growth.

## Discussion

Gene expression profiling has revolutionized our ability to understand the concerted and global response of the human transcriptome at the molecular level. Collectively, our data suggest that either expression or suppression of TIA-1 and/or TIAR proteins triggers pathway-specific and biologically coherent regulatory programs linked to an acute inflammation and cell proliferation-promoting transcriptome. They also reveal that TIA proteins can provide a novel regulatory layer of mechanisms for cross-talk between different pathways that coordinate cellular phenotypes such as metabolic homeostasis, cell survival and growth. Most importantly, we would like to indicate that our findings agree with and can explain at the molecular level previous observations based on mice models where suppression of TIA-1 expression promotes chronic inflammatory responses as well [32].

**Figure 7:**
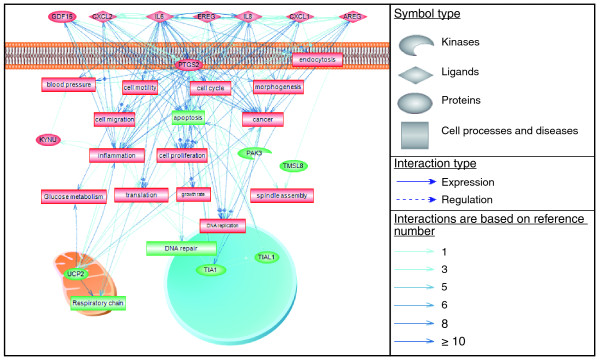
Interaction network associated with the gene cluster induced upon suppression of TIA proteins. Visualization of biochemical relationships between gene clusters up-regulated (red) and down-regulated (green) identified by microarray analyses were computed using the Pathway Studio (version 6.0) software and the 'ResNet Mammalian' database developed by Ariadne Genomics, Inc. [40]. The network topology illustrates positive (red boxes) and negative (green boxes)-regulated cell processes based on interactions after inactivation of TIA proteins. The nature of the regulators is depicted schematically as shown in the legend to the right. Continuous and dotted lines illustrate direct and indirect protein-protein interactions, respectively. The thickness of blue lines is proportional to the number of interactions reported in the literature.

A molecular hallmark associated with the depletion of TIA proteins comprises a gene signature including those encoding pro-inflammatory cytokines (*IL-1A *and *IL-6*), inflammatory chemokines (*CXCL1*, *CXCL2 *and *IL-8*), growth-stimulating factors (*AREG*, *EREG *and *GDF15*) and pro-angiogenic inducers (*PTGS2 *and *IL-8*) as well as the RAS oncogene family member *RAB40B*, regulators of cytoskeleton organization and biogenesis (*TMSL8 *and *PAK3*) and a mitochondrial modulator (*UCP2*). This gene cluster contains the ensemble of gene functions that may coordinately work to activate the acquisition of cell proliferation and anchorage-independent cell growth phenotypes observed in TIA-1/TIAR-depleted cells. Using a bioinformatic tool (the Pathway Studio software (version 6.0) and the 'ResNet Mammalian' database developed by Ariadne Genomics), a gene network based on biochemical data was created for experimentally determined interactions between clusters of up- and down-regulated genes associated with the depletion of TIA-1 and TIAR in HeLa cells (Figure 7; Additional data file 9). This network suggests a cellular scenario where silencing of these proteins leads to increased expression of a gene cluster encoding components of inflammation, angiogenesis and cell growth signaling, with a predicted enhancer effect on these pathways (Figure 7; Additional data file 9). This *in silico *simulation could integrate our results at a molecular level as well as previous observations on the importance of the cell-cell signaling, chemotaxis, adhesion, angiogenesis and immune responses mediated by interleukins, chemokines and growth-stimulating factors in molecularly connecting chronic inflammation and compensatory proliferation [33,34].

Why should inflammation and cell proliferation signaling result in a coherent transcriptional and post-transcriptional program of gene regulation targeting similar classes of genes? Promotion of cell proliferation and growth can be achieved through either abnormally activated or deregulated signaling pathways involved in cell cycle regulation or, quite often, abnormal growth signals outside the transformed cell, which are usually not considered. In this regard, TIA-1/TIAR target genes regulating proliferation include those encoding growth- and survival-stimulating factors, such as the epidermal growth factor receptor (EGFR)/pan-HER ligand epiregulin (EREG), the epidermal growth factor-like growth factor amphiregulin (AREG), and a member of the transforming growth factor-beta (TGF-beta) superfamily known as growth differentiation factor-15 (GDF15) (Figure 7). Moreover, the cellular processes and pathways promoting these growth-stimulating factors can be reinforced by IL-6 and IL-8, the chemokines CXCL1 and 2 and cyclooxygenase-2, which contribute to cell growth through self-sufficiency in growth signals, evasion of apoptosis, insensitivity to growth inhibitors as well as inflammatory and angiogenic potential responses [33-36].

These findings provide novel insights into the critical role of individual regulators such as TIA proteins to control, indirectly rather than directly, protein output from hundreds of target genes in a given cellular context. These regulators may impact physiological processes by regulating the concentrations of a few key cellular proteins that may be components of a single or functionally interrelated pathways. This is an interesting observation because it suggests that the molecular links that lead to gene expression control are intimately interconnected, pointing to a widespread role for TIA proteins in the negative regulation of transcriptional rates of the human genome. Thus, these proteins may function as adaptors or coupling factors to facilitate cross-talk between different layers that define the complex interactions involved in the regulation of the human transcriptome.

A close inspection of differentially expressed genes between control versus TIA-1/TIAR-depleted HeLa cells reveals the up-regulation of cell-cycle activators such as *CDC23 *and the down-regulation of cell-cycle suppressors such as *CHES1*, *MPHOSPH9*, *CCNB1 *(encoding cyclin B1), *ANAPC7*, *NASP *and *CDC7*, which could also account for the enhanced proliferation. Additionally, genes for cell-cycle controlling transcription factors - for example, *TFDP1*, *TFDP2 *and *E2F8 *- as well as other cell-cycle progression related factors - *UHRF1*, *BARD1*, *RABGAP1*, *PSRC1*, *PCGF6 *and *SEPT6 *- are also down-regulated in HeLa cells with TIA-1/TIAR-induced silencing (Additional data files 9 and Additional data files 10). To our knowledge, this is the first study to analyze, in parallel, global transcriptomic and phenotypic impacts after changes in the expression and/or function of TIA proteins.

What could be the molecular mechanism that mediates the changes in steady-state mRNA levels triggered by TIA proteins? We propose that, in addition to mediating some of the signaling effects of the cytokines, chemokines and growth-stimulating factors, the putative molecular mechanism may implicate the activation of basal transcriptional machinery through up-regulation of the gene *TAF5L *as well genes for nuclear transcription factors - for example, the *CREM *and *MAFF *families - together with the down-regulation of genes for transcription repressors, such as *PCGF2*, *NR2F2*, *SUDS3 *and *PCGF6 *(Additional data files 9 and Additional data files 10). Constitutive activation/repression of basal transcriptional machinery and/or selective transcription factors is an emerging hallmark of various transformed cell types [33-35]. In addition, they have specifically been implicated in the development of proliferative phenotypes through autocrine and paracrine mechanisms. Our TIA-1/TIAR silencing signature is associated with the up-regulation of critical NF-κB and CREM effectors, including *CXCL1*, *CXCL2*, *PTGS2*, *IL-6 *or *IL-8*, which have been implicated in cell proliferation and growth [33-35]. In summary, TIA-1/TIAR-depleted HeLa cells are characterized by a molecular signature indicative of global and constitutive activation of NF-κB and CREM downstream effector genes associated with cell proliferation. Presumably, the up-regulation of genes required for proliferation, survival and inhibition of apoptosis might be essential for anchorage-independent growth. These conjectures, however, will have to be proven by additional studies in order to determine the precise role of these genes in the phenotypes observed in this study.

Nevertheless, the coordinate expression of inflammation and/or growth-stimulating genes requires both transcriptional and post-transcriptional regulation [34]. Indeed, this gene coordination is also able to exert a positive effect on inflammation and cell proliferation or a positive feedback control on these pathways by regulating the expression of some of its components in TIA-1/TIAR-dependent regulatory events. These multifunctional proteins have been implicated in several steps of RNA dynamics and metabolism, including transcription, RNA splicing, RNA stability and translation, but little is known about the putative mechanisms and selectivity of transcriptional repression exerted by these proteins. It is important to note that these regulators have been localized on the carboxy-terminal domain of RNA polymerase II [2]; therefore, they could interact with basal transcription machinery and influence its activity. In addition, both proteins contain three RNA-binding motifs and are structurally close to heterogeneous ribonucleoproteins [5]. Consistently, both TIA proteins directly bind to RNA as well as to single- and double-stranded DNA [3-5]. It is tempting to speculate that TIA proteins could also interact with specific promoter sequences and/or with gene-specific/general transcription factors [3,4]. In this regard, it is more than reasonable to suggest that these proteins can carry out functions during transcription. Further, an increasing body of evidence indicates that transcription and RNA splicing processes are coupled [2].

Collectively, we postulate that, during transcription, TIA-1 and/or TIAR proteins can be recruited from the carboxy-terminal domain of RNA polymerase II to interact with genomic DNA sequences and/or basal transcription machinery and subsequently slow down RNA polymerase II and, therefore, the rate of transcription. Moreover, these proteins can perform triple functions during chromatin structure organization, transcription and splicing because they can potentially shuttle between DNA and RNA substrates and it has been demonstrated that factors affecting the rate of RNA polymerase II-mediated transcriptional elongation/initiation could influence alternative splicing patterns [2].

On the other hand, the prevalent role of these proteins as gene expression post-transcriptional modulators in the regulation of processes of alternative pre-mRNA splicing as well as their role in the stability and translation of specific cellular mRNAs is well-documented [8,12-14,22-24]. TIA proteins regulate the alternative pre-mRNA splicing of approximately 15% of alternative cassette human exons through binding to U-rich intronic elements, facilitating atypical 5' splice site recognition by U1 small nuclear ribonucleoproteins [6-10]. These proteins have also been well characterized as regulators of the stability and translation of cellular mRNAs in the cytosol. The proteins have been implicated in stress-induced translational arrest, colocalizing after stress with poly(A)^+ ^RNA in the cytoplasmic foci known as stress granules [15]. TIA-1 and TIAR are both able to bind to the 3'-untranslated regions of the stability/translation regulatory U- and AU-rich elements [5,11-14,16]. This aspect of TIA-1/TIAR function has recently been the focus of a broad and systematic analysis to identify mRNAs associated with both RNA-binding proteins [22-24]. However, it is interesting to note that no overlap is observed between gene targets reported to be repressed by TIA-1 at the translation level [22] and genes regulated by TIA1/TIAR at the alternative splicing level [8]. This suggests that TIA proteins regulate distinct subsets of genes at the splicing and translation levels. Indeed, TIA-1 and/or TIAR proteins might independently switch on/off molecular plasticity in gene regulation machineries such as transcription, alternative splicing, stability and/or translation to coordinate the establishment of the diversity of specific transcriptomes and proteomes that are required in complex cellular responses such as cell proliferation and growth. Thus, the roles of these regulators and further newly identified targets in these physiological processes deserve further investigation.

## Conclusions

The data presented in this manuscript illustrate specific changes in transcriptomic dynamics associated with cell-cell signaling, immuno-suppression, inflammation, angiogenesis, metabolism and cell proliferation-related mRNAs upon silencing of TIA-1 and/or TIAR proteins in HeLa cells. A gene cluster including cytokines, chemokines and growth-stimulating factors was identified as a molecular hallmark/signature associated with the depletion of TIA proteins. From a mechanistic viewpoint, TIA proteins could modulate transcriptional and post-transcriptional events associated with the control of target gene promoter basal activity and specific mRNA turnover. Additionally, the results also show that the knockdown of TIA-1 and/or TIAR proteins in HeLa cells triggers cell proliferation and anchorage-independent growth. *In silico *simulation of a biochemical interaction network incorporating up- and down-regulated target genes is consistent with a set of functions coherent with the different roles of several *in vivo *pathways to activate the acquisition of cell proliferation and anchorage-independent growth phenotypes detected after TIA-1/TIAR silencing. Collectively, our results argue that silencing of TIA proteins can trigger pathway-specific changes and affect genes with coherent functions that are important for cell proliferation and growth responses.

## Materials and methods

### Cell culture and RNA interference analysis

HeLa S2 cells were processed and RNA interference experiments were carried out as described previously [10,25-27].

### Transfections, luciferase and β-galatosidase assays

The human *PTGS2 *promoter [GenBank:AF276953.2] construct containing the full-length promoter sequence fused to a firefly luciferase reporter gene, named p2-1900 (-1,796, +104), was generated as described previously [30]. The human *IL-6 *gene promoter [GenBank:AF372214.2] construct containing a 651-bp fragment of the human promoter sequence was fused to a firefly luciferase reporter gene, named pIL-6-luc651, as described previously [31]. Both constructs were kindly provided by Dr Y Revilla. The β-galactosidase plasmid pcDNA3.1/*myc-His*/*lacZ *(Invitrogen, Carlsbad, CA, USA) was kindly provided by Dr J Berlanga. Control and TIA-1/TIAR-depleted HeLa cells, as described above, were co-transfected transiently with 50 and 500 ng of specific reporter *lacZ *and firefly luciferase plasmids, respectively, in six-well plates. The cells were incubated at 37°C for 16 h and then post-transfected cells were stimulated with DMSO or 20 ng/ml of PMA in DMSO. Six hours after stimulation, cells were lysed with 200 μl of cell culture lysis reagent (Promega, Madison, WI, USA) and microcentrifuged at 14,000 rpm for 5 minutes at 4°C, and 20 and 40 μl of each supernatant was used to determine firefly luciferase activity in a Monolight 2010 luminometer (Analytical Luminescence Laboratory, San Diego, CA, USA). β-Galactosidase activity was measured as described previously [37]. Luciferase activity was expressed as relative light units (RLU) per milligram of protein determined by the bicinchoninic acid method (Pierce, Rockford, IL, USA) and normalized to β-galactosidase-specific activity expressed as nmoles of hydrolyzed o-nitrophenyl-β-D-galactopyranoside (Sigma, St. Louis, MO, USA) per minute and milligram of protein at 37°C. Co-transfection experiments were performed in triplicate and the data are presented as the means of the ratio RLU/β-galactosidase, expressed as fold induction relative to the corresponding control values (means ± standard error of the mean).

### Proliferation and cell-cycle analyses

For cell growth analysis, post-transfected HeLa cells (2 × 10^4^) were seeded and collected for counting from triplicate at the indicated time points for 6 days. Cell proliferation was also quantified by measuring the conversion of methyl thiazolyl tetrazolium into DMSO-soluble formazan by living cells, with absorbance measured at 560 nm using a spectrophotometer. Cell-cycle analysis was carried out by flow cytometry after propidium iodide staining.

### Anchorage-independent growth assays

Soft agar colony formation was assayed by plating in duplicate 1 × 10^3 ^cells in 0.35% agarose in 10% fetal calf serum containing media on a 0.8% agarose base in six-well plates. After 14 days, the resulting colonies were stained with 0.01% crystal violet solution, counted and photographed under a light microscope at 100× magnification.

### Preparation of cell extracts and western blot analysis

Whole-cell extracts were performed and processed as described previously [25]. Immunoblots were carried out using the following antibodies: anti-U2AF65 (MC3; provided by J Valcárcel); anti-PTGS2 (provided by MA Íñiguez), anti-TIA-1, anti-TIAR and anti-HuR (Santa Cruz Biotechnology, Santa Cruz, CA, USA), anti-Mcl-1 (Biomol, Farmingdale, NY, USA) and anti-α-tubulin (Sigma, St. Louis, MO, USA).

### RNA purification, semiquantitative and quantitative RT-PCR analyses and *in vivo *RNA decay determination

Cytoplasmic RNA isolations and semiquantitative RT-PCR analysis were carried out as described previously [10,25-27]. The expression of selected genes was assessed independently by quantitative RT-PCR as described in [37]. A list of primer pairs is given in Additional data file 11. RNA turnover experiments were performed by adding actinomycin D (5 μg/ml) to the growth medium.

### Transcriptome analysis

RNA quality checks, amplification, labeling, hybridization with Human Genome U133 Plus 2.0 Array Chips (approximately 55,000 transcripts; Affymetrix Inc., Santa Clara, CA, USA) and initial data extraction were performed at the Genomic Array Core Facility in Scientific Park, Madrid at the Universidad Complutense de Madrid [38]. Comparison of multiple cDNA array images (three independent experiments per biological condition tested) was carried out by using an average of all of the gene signals on the array (global normalization) to normalize the signal between arrays (Additional data file 1). Heat map and sample clustering plots as well as GO and KEGG database analyses were performed using software programs provided by Integromics™ [39]. Networks of biochemical relationships between gene clusters identified as up-regulated and down-regulated by microarray analysis were created using the Pathway Studio software (version 6.0) and the 'ResNet Mammalian' database developed by Ariadne Genomics, Inc. (Rockville, MD, USA) [40]. Microarray data have been deposited in the EMBL-EBI ARRAY EXPRESS database [41] and are accessible through the ArrayExpress accession number [ArrayExpress:E-MTAB-93].

### Statistical analysis

Represented values are shown as means ± standard error of the mean. Differences were tested for significance by means of the Student's *t*-test. TIA-1/TIAR siRNA effects on HeLa cells were compared with respect to the control siRNA transfection. A probability level *P *< 0.05 was considered significant.

## Abbreviations

AREG: amphiregulin; DMSO: dimethyl sulfoxide; EREG: pan-HER ligand epiregulin; GDF: growth differentiation factor; GO: Gene Ontology; IL: interleukin; KEGG: Kyoto Encyclopedia of Genes and Genomes; NF: nuclear factor; RLU: relative light units; siRNA: small interfering RNA; TIA-1: T-cell intracellular antigen-1; TIAL1/TIAR: TIA-1 like/related protein.

## Authors' contributions

JMI conceived the research and designed all the experiments. JMI, RR and JA carried out the experiments presented in this paper. RR, JA and JMI wrote the paper. All authors provided feedback and approved the final manuscript.

## Additional data files

The following additional data are available with the online version of this paper: variability of data as function of the mean (MvA), RNA digestion, raw and normalized data box plots (Additional data file 1); summary of differentially expressed genes in TIA-1 and TIAR-depleted HeLa cells (Additional data file 2); summary of GO and KEGG database analyses of microarray-predicted genes from TIA-1 and TIAR-depleted HeLa cells (Additional data file 3); summary of differentially expressed genes in TIA-1-depleted HeLa cells (Additional data file 4); summary of differentially expressed genes in TIAR-depleted HeLa cells (Additional data file 5); global mRNA expression patterns in TIA-1 or TIAR-depleted HeLa cells (Additional data file 6); summary of GO and KEGG database analyses of microarray-predicted genes from TIA-1-depleted HeLa cells (Additional data file 7); summary of GO and KEGG database analyses of microarray-predicted genes from TIAR-depleted HeLa cells (Additional data file 8); global network of genes modulated after TIA-1 and TIAR silencing (Additional data file 9); list of genes up- and down-regulated after TIA-1/TIAR silencing that function as transcription and/or cell-cycle modulators (Additional data file 10); list of primer pair sequences used to validate microarray-predicted genes by real-time PCR analysis (Additional data file 11).

## Supplementary Material

Additional data file 1 **(a) **MvA plots. From left to right: MvA plots derived from high density oligonucleotide arrays to define gene expression profiles from control (C_1-3.CEL files) and TIA-1 (1_1-3.CEL files), TIAR (R_1-3.CEL files) or TIA-1 plus TIAR (1+R_1-3.CEL files)-depleted HeLa cells. This kind of plot is used to diagnose experimental problems between different biological replicates. The ratio between array signal intensities is illustrated on the y-axis (M) and intensity average is represented on the x-axis (A). In this type of plot, if there are no systematic differences between biological replicates, the dot map of each array is concentrated on the axis M = 0. In all cases documented in our experiments, there was no a significant variability between different biological samples. **(b) **RNA digestion plots. RNA degradation mainly started from the 5'-end and, therefore, a greater loss of signal intensity occurred with the probes that hybridize to the mRNA 5'-end than with complementary probes close to the mRNA 3'-end. RNA degradation plots illustrate mRNA intensity values versus the function of the positions corresponding to the probes in the 5'-3' orientation. Each graphic on the plot illustrates one array and the slope indicates the RNA degradation level corresponding to each hybridized sample. Overall, a higher slope indicates that more hybridized sample was degraded. This kind of plot is likely to detect significant differences between hybridized samples. Our results show that all samples behaved similarly, suggesting that there are no significant differences between biological samples analyzed. **(c) **Box plots of crude (to the left) and normalized (to the right) data analyzed by the quantile technique. The Data_rma.txt file submitted to the ArrayExpress database contains processed data using the Robust Multichip Average method [28]. Every color indicates a characteristic group of biological samples analyzed. Click here for file

Additional data file 2 List of the significantly up- and down-regulated genes in TIA-1 and TIAR-depleted HeLa cells analyzed by using Integromics™ software [39]. The studied gene parameters are as follow. Affymetrix Probe Set ID: probe-set identifier of the Affymetrix database [42]. Average Fold Change: mean value of fold change, expressed as base 2 logarithm, for each gene between the compared situations. RP Value Up: probability for a specific gene to be up-regulated. Expected UP: expected value for a certain RP Value Up (it represents the average number of times that an equal or better RP value appears in randomized experiments). FDR Up: expected percentage of false positives for up-regulated genes. RP Value Down: probability for a specific gene to be down-regulated. Expected Down: expected value for a certain RP Value Down. FDR Down: expected percentage of false positives for down-regulated genes. Venn Up: shows in which of the three compared situations (control versus TIA-1 (T1), control versus TIAR (T2) or control versus TIA-1 and TIAR (T3)) the gene is up-regulated. Venn Down: shows in which of the three compared situations as above the gene is down-regulated. UniGene ID: identification number of each gene in the UniGene database [43]. Gene Title: gene name. Gene Symbol: acronym of each gene. Chromosomal Location: gene location on the chromosome. Entrez Gene: probe-set identifier in the Entrez Gene database [44]. SwissProt: protein accession number in SwissProt database [45]. OMIM: accession number in Online Mendelian Inheritance in Man, a database for human genes and genetic disorders [46]. GO Biological Process, GO Cellular Component, GO Molecular Function: accession number and description of the assigned GO in biological process, cellular component and molecular function categories [47]. Pathway: accession number and gene pathway assigned in the biologically represented pathways of the GenMAPP database [48]. InterPro: accession number in the InterPro database and description of protein families and domains [49]. Click here for file

Additional data file 3 Analyses of KEGG pathways and GO terms corresponding to the biological process category were applied to differentially expressed genes in TIA-1 and TIAR knockdown HeLa cells. Calculated *P*-values for each pathway or category are shown. All analyses were performed using Integromics™ software [39]. Click here for file

Additional data file 4 List of the up- and down-regulated genes in TIA-1-depleted HeLa cells. The analyzed databases were the same as in Additional data file 2. Click here for file

Additional data file 5 List of the up- and down-regulated genes in TIAR-depleted HeLa cells. The analyzed databases were the same as in Additional data file 2. Click here for file

Additional data file 6 **(a-h) **Genome-wide microarrays were performed with TIA-1 or TIAR-depleted HeLa cells as in Figure 2. (a-d) Graphic pie representations of the distribution of up- (a, b) and down-regulated (c, d) genes (*P *< 0.01) in TIA-1 (a, c) or TIAR (b, d) knockdown HeLa cells using the biological process category of the GO database. (e-h) Graphic pie representations of the distribution of up- (e, f) and down-regulated (g, h) genes (*P *< 0.05) in TIA-1 (e, g) or TIAR (f, h) knockdown HeLa cells using the KEGG pathway database. In all cases (a-h), percentages indicat the portion of total genes that are associated with the biological functions and pathways shown. Click here for file

Additional data file 7 Analysis of KEGG pathways and GO terms corresponding to the biological process category was applied to differentially expressed genes in TIA-1 knockdown HeLa cells. Calculated *P*-values for each pathway or category are shown. All analyses were performed using Integromics™ software [39]. Click here for file

Additional data file 8 Analysis of KEGG pathways and GO terms corresponding to the biological process category was applied to differentially expressed genes in TIAR knockdown HeLa cells. Calculated *P*-values for each pathway or category are shown. All analyses were performed using Integromics™ software [39]. Click here for file

Additional data file 9 Biochemical relationships between gene clusters up-regulated (red) and down-regulated (green) as identified by microarray analyses were created using Pathway Studio software (version 6.0) from Ariadne Genomics [40]. Click here for file

Additional data file 10 Summary of differentially expressed genes in TIA-1 and TIAR-depleted HeLa cells that function as transcription and/or cell cycle regulators. The set of studied gene parameters are as follow. Gene Symbol: acronym of each gene. Gene Title: gene name. Fold Change: mean value of fold change for each gene between control and TIA-1 and TIAR-depleted HeLa cells. GO Biological Process: accession number and description of the assigned GO in the biological process category [47]. Functions: summary of the more important roles associated with the list of transcription and/or cell-cycle-related genes. Click here for file

Additional data file 11 The primer sequences were designed using the Universal ProbeLibrary Assay Design Center software from Roche [50] and yielded only one amplified product using a BLAST search [51]. The analyzed genes were: *APPBP2*, Amyloid beta precursor protein binding protein 2; *AREG*, Amphiregulin; *β-actin*, Actin, beta; *CD24*, CD24 molecule; *CXCL1*, Chemokine (C-X-C motif) ligand 1; *CXCL2*, Chemokine (C-X-C motif) ligand 2; *EREG*, Epiregulin; *FASTK*, Fas-activated serine/threonine kinase; *GDF15*, Growth differentiation factor 15; *IL-1A*, Interleukin 1, alpha; *IL-6*, Interleukin 6; *IL-8*, Interleukin 8; *KYNU*, Kynureninase (L-kynurenine hydrolase); *MMP2*, Matrix metallopeptidase 2; *MTFR1*, Mitochondrial fission regulator 1; *OPN1*, Opsin 1 short-wave-sensitive; *PAK3*, p21 protein (Cdc42/Rac)-activated kinase 3; *PTGS2*, Prostaglandin-endoperoxide synthase 2; *RAB40B*, RAB40B, member of the RAS oncogene family; *TFDP2*, Transcription factor Dp-2; *TIA-1*, T-cell intracellular antigen 1; *TIAR*, TIA-1 related protein; *TIMP2*, TIMP metallopeptidase inhibitor 2; *TMSL8*, Thymosin like-8; *TNFSF10*, Tumor necrosis factor superfamily, member 10; and *UCP2*, Mitochondrial uncoupling protein 2. Click here for file
