# Supplementary material for: Depletion of T-cell intracellular antigen proteins promotes cell proliferation
Source: Genome Biol. 2009 Aug 26;10(8):R87. doi: 10.1186/gb-2009-10-8-r87 (PMC2745768; doi:10.1186/gb-2009-10-8-r87)
Supplement: Additional data file 11 — The primer sequences were designed using the Universal ProbeLibrary Assay Design Center software from Roche [50] and yielded only one amplified product using a BLAST search [51]. The analyzed genes were: APPBP2, Amyloid beta precursor protein binding protein 2; AREG, Amphiregulin; β-actin, Actin, beta; CD24, CD24 molecule; CXCL1, Chemokine (C-X-C motif) ligand 1; CXCL2, Chemokine (C-X-C motif) ligand 2; EREG, Epiregulin; FASTK, Fas-activated serine/threonine kinase; GDF15, Growth differentiation factor 15; IL-1A, Interleukin 1, alpha; IL-6, Interleukin 6; IL-8, Interleukin 8; KYNU, Kynureninase (L-kynurenine hydrolase); MMP2, Matrix metallopeptidase 2; MTFR1, Mitochondrial fission regulator 1; OPN1, Opsin 1 short-wave-sensitive; PAK3, p21 protein (Cdc42/Rac)-activated kinase 3; PTGS2, Prostaglandin-endoperoxide synthase 2; RAB40B, RAB40B, member of the RAS oncogene family; TFDP2, Transcription factor Dp-2; TIA-1, T-cell intracellular antigen 1; TIAR, TIA-1 related protein; TIMP2, TIMP metallopeptidase inhibitor 2; TMSL8, Thymosin like-8; TNFSF10, Tumor necrosis factor superfamily, member 10; and UCP2, Mitochondrial uncoupling protein 2. [file gb-2009-10-8-r87-S11.pdf]

Gene name	Forward primer	Reverse primer	Product length (bp)
APPBP2	5'-AAGTACTTCCAGCTGACTCAGGTGTT-3'	5'-GACGTCTGTAGTTCCGCTGCT-3'	71
AREG	5'-ACTCGGCTCAGGCCATTATG-3'	5'-AAAATGGTTCACGCTTCCCA-3'	71
β-actin	5'-AAAGACCTGTACGCCAACAC-3'	5'-GTCATACTCCTGCTTGCTGA-3'	219
CD24	5'-AGAACCTGGTCCTAAGCCTAAAAGT-3'	5'-CTGTGTTTCGAGGCAGTTGTAAA-3'	71
CXCL1	5'-TCCTGCATCCCCCATAGTTA-3'	5'-CTTCAGGAACAGCCACCAGT-3'	105
CXCL2	5'-CGCCCATGGTTAAGAAAATC-3'	5'-AGGAACAGCCACCAATAAGC-3'	93
EREG	5'-CTCTGCCTGGGTTTCCATCTT-3'	5'-TCTCCTGGGATACATGATGGAATC-3'	71
FASTK	5'-TTGCAGTCTTACTTGGCTTTCCA-3'	5'-AGGCGGAGCCTTCGCT-3'	73
GDF15	5'-AGTTGCGGAAACGCTACGAG-3'	5'-GGTGTTCGAATCTTCCCAGCT-3'	71
IL-1A	5'-AGAGGAAGAAATCATCAAGCCTAGGT-3'	5'-TCATAAAGTTGTATTTCACATTGCTCAG-3'	71
IL-6	5'-CGGGAACGAAAGAGAAGCTCTAT-3'	5'-CGAAGGCGCTTGTGGAGA-3'	72
IL-8	5'-AGAGCTCTGTCTGGACCCCA-3'	5'-TCTCAGCCCTCTTCAAAAACTTCT-3'	71
KYNU	5'-TCTGTGGATTCCGAATTTCAAA-3'	5'-AAAGATCTCTAAACTAGCATGCAAGGA-3'	71
MMP2	5'-ACAAATTCTGGAGATACAATGAGGTG-3'	5'-GGCATCTGCGATGAGCTTG-3'	71
MTFR1	5'-GTAAAACTTCGGTCAGTGAAGAGGT-3'	5'-CAGCAGGGTCAGTAGCATCCA-3'	73
OPN1	5'-GATACTTCGTCTTCGGTCGCC-3'	5'-TGTAACCAGACCTGCTACAGTGC-3'	71
PAK3	5'-CCAGGCTTCGCTCTATCTTC-3'	5'-GATCTCTGGGCGCTCTTTC-3'	74
PTGS2	5'-TATAAGTGCGATTGTACCCGGAC-3'	5'-ATTCTTGTCAAAAATTCCGGTGTT-3'	71
RAB40B	5'-GGGCATCGACTACAAGACG-3'	5'-AATCTTCCCTGGCCTGAAGT-3'	87
TFDP2	5'-ACCCCAGTCAAGTGTAAACCAAG-3'	5'-GGCCAGGAACTGCCCAG-3'	73
TIA-1	5'-GCCCAAGACTCTATACGTCGGTAACC-3'	5'-GGTGCAAAAGCAGCTTTTATATCTTC-3'	336
TIAR	5'-ATGATGGAAGACGACGGGCAGCCCCGGACTC-3'	5'-TCTGGACTCAAATCCCCAACAAACACATGG-3'	320
TIMP2	5'-CGACATTTATGGCAACCCTATCA-3'	5'-TCTCAGGCCCTTTGAACATCTT-3'	74
TMSL8	5'-CCTCAAAGGAAACTATCCAGCAA-3'	5'-TGTTGGGAGGCGATCCC-3'	71
TNFSF10	5'-TGCTCCTGCAGTCTCTCTGTGT-3'	5'-GTCCTGCATCTGCTTCAGCTC-3'	71
UCP2	5'-ACGGGACACCTTTAGAGAAGCTT-3'	5'-CTGCCGGAATCCGGC-3'	71
